# Cerebral oximetry and brain death in the ICU: data from seven cases

**DOI:** 10.1186/cc10901

**Published:** 2012-03-20

**Authors:** N Billet, I Meex, M Vanderlaenen, R Heylen, W Boer, C De Deyne, FV Jans

**Affiliations:** 1 Ziekenhuis Oost-Limburg, Genk, Belgium; 2 University of Hasselt, Diepenbeek, Belgium

## Introduction

Cerebral oximetry, using near-infrared spectroscopy to measure cerebral tissue oxygen saturation (SctO_2_), is being increasingly used in the ICU. We hypothesized that if a patient becomes brain dead in the ICU, this must be reflected in SctO_2 _values. This might help in the timing of invasive procedures such as angiography, sometimes necessary in the confirmation of brain death.

## Methods

We retrospectively analyzed the cerebral oximetry data of seven patients with severe TBI or diffuse cerebral edema who evolved to brain death while being treated in the ICU. Absolute SctO_2 _values were continuously measured with ForeSight technology (Casmed) with sensors applied bilaterally to the forehead.

## Results

Three patients (one TBI and two SAH) with continuous ICP and SctO_2 _monitoring suffered, despite maximal medical treatment, a sudden (over 1 to 3 hours) increase in mean ICP from 32 mmHg to 91 mmHg (equalization of ICP and MAP). Over the same time period, a parallel decrease in mean SctO_2 _from 71% to 54% ICP was observed. One patient (cerebral edema after asphyxia) had continuous EEG and SctO_2 _monitoring: a sharp decrease in SctO_2 _from 67% to 56% over 30 minutes was accompanied by an increase in suppression ratio from 70% to 100%. The absence of cerebral blood flow was confirmed by CT angiography. One patient (cerebral edema after prolonged CPR) had only SctO_2 _measurement for cerebral monitoring: during his stay in the ICU, there was a sudden decrease in SctO_2 _from 64% to 54% over a 90-minute period. Shortly after this, the pupils became dilated and fixed. Brain death was confirmed by full EEG. Three brain-dead patients with documented absence of cerebral blood flow were monitored for SctO_2 _during subsequent organ donation procedure: SctO_2 _remained at a mean value of 59% during the procedure, and fell sharply only at the onset of circulatory arrest to reach a stable value of 25%.

## Conclusion

In this small cohort of patients, the onset of brain death was accompanied in all cases by a sharp and large decrease in SctO_2 _from 67% to 55% (mean, *n *= 5) and remained stable. SctO_2 _values only reached minimal values (25%, *n *= 3) at complete circulatory arrest. Our data suggest that SctO_2 _measurement may be helpful in the timing of the diagnosis of brain death, especially in those patients without ICP or continuous EEG monitoring.

